# Recommended Intake of Key Food Groups and Cardiovascular Risk Factors in Australian Older, Rural-Dwelling Adults

**DOI:** 10.3390/nu12030860

**Published:** 2020-03-23

**Authors:** Alice J. Owen, Michael J. Abramson, Jill F. Ikin, Tracy A. McCaffrey, Sylvia Pomeroy, Brigitte M. Borg, Caroline X. Gao, David Brown, Danny Liew

**Affiliations:** 1 School of Public Health and Preventive Medicine, Monash University, 553 St Kilda Rd, Melbourne, VIC 3004, Australia; michael.abramson@monash.edu (M.J.A.); jill.blackman@monash.edu (J.F.I.); sylvia.pomeroy@monash.edu (S.P.); brigitte.borg@monash.edu (B.M.B.); caroline.gao@monash.edu (C.X.G.); david.brown@monash.edu (D.B.); danny.liew@monash.edu (D.L.); 2 Department of Nutrition, Dietetics and Food, Monash University, Melbourne, VIC 3168, Australia; tracy.mccaffrey@monash.edu; 3 Respiratory Medicine, Alfred Hospital, Melbourne, VIC 3004, Australia

**Keywords:** diet quality, cardiometabolic risk, sugar-sweetened beverages, food groups

## Abstract

This study examined the relationship between diet quality scores and cardiometabolic risk factors in regionally-dwelling older Australian adults with increased cardiovascular risk. This study was a cross-sectional analysis of demographic, anthropometric, and cardiometabolic risk factor data from 458 participants of the Cardiovascular Stream of the Hazelwood Health Study. Participants completed a 120 item semi-quantitative food frequency questionnaire. Multivariable linear regression adjusting for age, sex, smoking, physical activity, education, diabetes, and body mass index was used to examine the relationship between diet and cardiometabolic risk factors. Mean (SD) age of participants was 71 (8) years, and 55% were male. More than half of men and women did not meet recommended intakes of fibre, while 60% of men and 42% of women exceeded recommended dietary sodium intakes. Higher diet quality in terms of intake of vegetables, grains, and non-processed meat, as well as intake of non-fried fish, was associated with more favourable cardiometabolic risk profiles, while sugar-sweetened soft drink intake was strongly associated with adverse cardiometabolic risk factor levels. In older, regionally-dwelling adults, dietary public health strategies that address whole grain products, vegetable and fish consumption, and sugar-sweetened soft-drink intake may be of benefit in reducing cardiometabolic risk.

## 1. Introduction

Cardiometabolic diseases remain a major cause of mortality and morbidity across the globe, with high blood pressure, smoking, elevated plasma glucose, and high body mass index (BMI, kg/m^2^), the top four risk factors for attributable disability-adjusted life years [1]. Dietary intake is a well-known risk factor for non-communicable diseases, and the Global Burden of Disease Study recently estimated that 11 million deaths were attributable to dietary risk factors in 2017 [2]. In examining the mortality attributable to poor quality diet, it was estimated that the top five risks were diets high in sodium, low in whole grains, low in fruits, low in nuts and seeds, and low in vegetables [2]. However, limited availability of geographically representative data remains a barrier to a clearer understanding of dietary risks, and to the development of effective local interventions to reduce the cardiometabolic disease risk conferred by inadequate dietary intake.

Age-and sex-specific dietary intake guidelines for maintaining health have been developed in many countries, including Australia, largely on the basis of findings from observational and prospective cohort data [3]. However, for many foods and nutrients, adherence to these guidelines is low [4]. In the 2011/12 Australian Health Survey, less than 1 in 25 adults met recommended guideline intakes of vegetables and legumes [4]. While among those aged 51–70 years, only 5% of men and less than 1% of women met guideline-recommended intakes of dairy foods/alternatives [4]. In addition, there was evidence that dietary risk factors may be influenced by socioeconomic and geographic factors, with rural residential status, education, and other socioeconomic status markers previously reported to be related to dietary quality and fibre intake [5,6,7]. There is also some evidence of gender differences in adherence to dietary quality and guideline adherence in non-metropolitan areas [6].

This study aimed to characterise diet quality in two rural Australian towns with a high burden of cardiovascular disease [8] and to examine associations with cardiometabolic risk factors using a recently developed dietary quality score [9], which allowed key food group diet quality to be explored.

## 2. Materials and Methods

### 2.1. Participants

**Figure 1:**
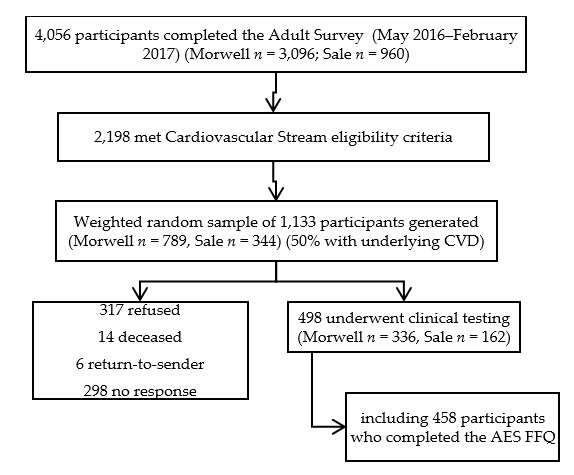
Flow diagram of participant recruitment. CVD = cardiovascular disease, AES = Australian Eating Survey, FFQ = food frequency questionnaire

The sample for this study comprised study participants from the Cardiovascular Stream of the Hazelwood Health Study, who additionally agreed to complete a dietary survey. Recruited between October 2017 and May 2018, Cardiovascular Stream participants were drawn from a weighted random sample of 1133 people who had previously completed the Hazelwood Health Study Adult Survey [10], lived in the rural Victorian towns of Morwell or Sale, and were males aged 55–89 years or females 60–89 years (Figure 1). Those who identified any underlying cardiovascular condition on the Adult Survey were oversampled. Years of education was captured as the highest educational qualification and classified as up to year 10, upper secondary (to year 11–12), trade certifications, or university/tertiary education. Residential area-related socioeconomic status was determined through the linkage of participant residential postcode with the Index of Relative Socioeconomic Advantage and Disadvantage (IRSAD) [11].

### 2.2. Measures

#### 2.2.1. Cardiometabolic Risk Factors

Participants attended a clinic during which a number of health assessments were made. Anthropometric measures included height and weight, from which body mass index (BMI) was calculated. Hip and waist circumference were also measured. Height (to nearest 0.1 cm) was measured using a wall-mounted stadiometer, waist circumference (to nearest 0.1 cm) was measured at the midpoint of the last palpable rib and top of the hip bone, and hip circumference (to nearest 0.1 cm) was taken as the point of maximum circumference around the buttocks. Weight was measured on calibrated standing scales to the nearest 0.1 kg. World Health Organization criteria were used to categorise BMI into overweight (BMI from ≥25 to <30 kg/m^2^) and obesity (BMI ≥30 kg/m^2^) [12]. Other cardiometabolic risk factors assessed were blood pressure, heart rate, plasma cholesterol, haemoglobinA1c, and creatinine, from which the estimated glomerular filtration rate (eGFR) was calculated as a marker of renal function. Blood pressure (mmHg) was measured in a seated position three times using a digital automatic blood pressure monitor (Omron, Matsusaka, Japan) with a one-minute rest between readings. The average of the last two measurements was used in the analysis. A non-fasting blood sample was taken for measurement of plasma cholesterol and haemoglobinA1c (HbA1c). eGFR was calculated using the Chronic Kidney Disease Epidemiology Collaboration (CKD-EPI) formula, which used age, gender, and blood creatinine to estimate renal function (expressed in mL/min/1.73 m^2^) [13]. The self-reported behavioural risk factors collected were smoking status, history of diabetes and cardiovascular disease and physical activity. The presence of diabetes was determined by self-reported doctor diagnosis, use of diabetes medications or HbA1c ≥ 6.5%. History of cardiovascular disease was self-reported doctor diagnosis. Smoking status was determined by self-reported smoking of at least 100 cigarettes, or a similar amount of tobacco, in a participant’s lifetime, and reporting of current or former smoking. Self-reported physical activity was assessed using the validated Active Australia survey, an eight-item questionnaire that captured information on time spent undertaking walking, household physical activity, vigorous physical activity, and moderate physical activity in the past week [14]. Participants were considered to have engaged in ‘adequate physical activity’ if they reported having undertaken any vigorous physical activity or at least 150 min of moderate physical activity over the previous seven days.

#### 2.2.2. Dietary Intake Assessment

The Australian Eating Survey Food Frequency Questionnaire (AES FFQ), a 120 item semi-quantitative FFQ previously validated in community-dwelling adults aged 30–70 years [9], was used to assess dietary intake. The dietary assessment was a voluntary component of clinic visits, and in some cases, completion was not undertaken due time constraints or participant preference. Nutrient intakes were computed against the AusNut Database [15]. The Australian Recommended Food Score (ARFS), a diet quality index that captures the dietary quality of key food groups, was calculated from the AES FFQ, as previously described [9]. The ARFS is computed as a total score, as well as subscales relating to intakes of vegetables, fruit, meat, non-meat protein, grains (breads and cereals), dairy, water, and spreads/sauces. Reported food items within the food sub-groups were awarded points for frequency of consumption based upon Australian national dietary guidelines, and the ARFS scores were calculated by summing the points for each item, as previously described [9]. To determine proportions with inadequate intakes of key macro- and micronutrients, estimated intakes were compared to age- and sex-specific estimated average requirements (EAR) or, in the case of sodium and potassium, suggested dietary target (SDT) and adequate intake (AI), respectively [16].

### 2.3. Statistical Analysis

Data analysis was undertaken using the Statistical Program for Social Sciences (IBM SPSS; Armonk, NY, USA), version 25. Standard descriptive statistics were used to examine cohort characteristics following assessment of normality. Associations between variables were explored using Pearson and Spearman’s correlations, as appropriate. Multivariable linear regression was used to examine the associations between diet quality indices and cardiovascular risk factors. Interactions between gender and dietary predictor variables were assessed by the inclusion of gender–diet interaction terms in models. Associations were initially examined in a minimally-adjusted model (Model 1, adjusted for age and sex), followed by multivariable-adjusted modelling adjusted for age, sex, smoking, physical activity, education, and diabetes for models examining waist:hip, and the addition of BMI as a covariate for all other risk factors (Model 2). History of cardiovascular disease, antihypertensive use, and lipid lowering therapy use were tested for inclusion in models. Lipid lowering therapy use was significant in models examining total cholesterol and was included in final models for this variable. For linear regression analyses examining fish and beverage intakes, Model 3 additionally included total dietary quality (ARFS total). To adjust for potential misreporting, regression analyses were repeated, excluding those with total dietary energy below 2000 kJ or above 15,000 kJ [17].

### 2.4. Ethics

The Hazelwood Health Study Cardiovascular Stream protocol was reviewed and approved by the Monash University Human Research Ethics Committee (project#1078). All participants provided informed written consent to participate, and this research was conducted in accordance with the Declaration of Helsinki.

## 3. Results

A flow diagram showing recruitment from the Adult Survey through to the Cardiovascular Stream is shown in Figure 1. From 498 Cardiovascular Stream participants who attended the clinic, 458 completed the AES FFQ and were included in the analysis.

### 3.1. Participant Characteristics

The mean (SD) age of participants was 71 (8) years, and over 55% were male (Table 1). There was a high prevalence of overweight and obesity in the cohort, with 83% of men and 77% of women having a BMI ≥25 kg/m^2^, and 46% of men and 48% of women having a BMI ≥30 kg/m^2^. Based on residential area, two-thirds of participants were categorised in the first (most disadvantaged) quintile of the IRSAD [11] (Table 1). Eighteen per cent of participants reported a history of diabetes, and 49.3% reported a history of cardiovascular disease (Table 1).

### 3.2. Dietary Intake

Mean diet quality (assessed by the ARFS) was 29.3/73 for men and 32.8/73 for women. Compared with men, women reported higher age- and education-adjusted diet quality scores for ARFS total (*p* < 0.001) and for some specific food groups: ARFS vegetables (*p* < 0.001), ARFS fruit (*p* = 0.004), and ARFS dairy (*p* = 0.01) (Table 2). When compared to Nutrient Reference Values for Australians [16], intakes of protein, iron, and vitamins were mostly adequate, while more than half of participants reported inadequate intake of fibre and more than 40% reported inadequate folate and calcium intakes. A greater proportion of men than women reported above recommended intakes of sodium and below recommended intakes of potassium (Table 3).

Consumption of sugar-sweetened beverages (soft drinks and cordials) at least once per week was reported by 32.0% of men and 19.3% of women (Table 2), with men having a substantially higher proportion of total dietary energy derived from sweetened drinks compared to women 3.5% vs. 1.9%, respectively (*p* < 0.001). There was also a sex-difference in fresh fish intake (*p* = 0.01), with women more likely to consume fresh fish at least once a week (Table 2).

### 3.3. Associations between Diet Quality and Cardiometabolic Risk Factors

Key cardiometabolic risk factors associated with diet quality were examined (Table 4). No associations were observed between AFRS total or component scores and HbA1c, (low density lipoprotein) LDL-cholesterol, blood pressure, or heart rate. There were no significant interactions between gender and dietary predictors evident in the models, except in the cases of the eGFR and ARFS score for alternate sources of protein (*p* = 0.006), and soft drinks and HbA1c (*p* = 0.012). In both age- and gender-adjusted models, and models adjusting for comorbidities, there was strong evidence that ARFS total, fruit, and grain scores were negatively associated with central adiposity (waist:hip) (Table 4). Higher quality of vegetable and meat intakes were positively associated with higher (high density lipoprotein) HDL-cholesterol. The dairy score was negatively associated with total cholesterol in minimally and fully adjusted models (Table 4), but there was no association between total cholesterol and percentage of total energy derived from all dairy products (results not shown). The association between ARFs alternate protein sources was significant in women (unstandardised beta coefficient B = 2.024, 95% CI: 0.677, 3.371, *p* = 0.003) but not men.

Intake of fresh fish was associated with higher eGFR, while intake of crumbed/battered fish was associated with a higher waist:hip ratio (Table 5). Canned fish intake was associated with higher HDL-cholesterol and lower HbA1c, although this finding was not significant when potential dietary misreporters were excluded (Table 5).

In both minimally and fully adjusted models, the proportion of dietary energy derived from sugar-sweetened drinks was positively associated with central adiposity and HbA1c but negatively associated with HDL-cholesterol and eGFR (Table 6). No associations between sugar-sweetened beverage intake and LDL or total cholesterol were observed. After adjusting for behavioural risk factors, demographic factors, and overall diet quality, each additional daily consumption of soft drink was associated with a 0.06 mmol/L decrease in HDL-cholesterol, and a 0.18 unit increase in HbA1c (Table 6). However, there was a soft drink–gender interaction seen for HbA1c, with the association significant in men (B = 0.166, 95% CI: 0.085, 0.245, *p* < 0.001) but not women. No associations were observed between cardiometabolic risk markers and other discretionary sweet food consumption, such as confectionery or sweet baked goods.

## 4. Discussion

In this cohort of older adults living in a regional area of south-eastern Australia, diet quality was on average lower than that previously reported for another Australian regionally-located cohort [9]. The Global Burden of Disease study has identified key dietary risk factors for non-communicable disease mortality as diets high in sodium, low in whole grains, and low in fruits and vegetables [2], all dietary risk patterns evident in this cohort. Intake of sugar-sweetened beverages was adversely associated with cardiometabolic risk factors, while intake of fresh and canned fish was beneficially associated with cardiometabolic risk factors. Prevalence of overweight and obesity in our cohort was higher than previously reported for those aged 65–74 years in Australia, which in 2015 was 80% in men and 69% in women [18]. The proportion of our cohort with diabetes was similar to that previously reported for Australians for aged 65 years and above (18.1% in this cohort compared to 17.4% in the 2014/15 Australian National Health Survey) [19]. Intake of sodium by this cohort was comparable to that reported in the Australian Health Survey (AHS) for women (1972 mg/d for 51–70-year-old women in AHS versus 1984 mg/d herein) but slightly lower in this cohort than population data previously reported for 51–70-year-old men in Australia (2510 mg/d in AHS vs. 2329 mg/d herein) [20]. However, dietary survey methods for assessing sodium intake are well-recognised to under-report sodium intake when compared to 24 h urinary sodium excretion studies [21].

Historically, dietary epidemiology has had a strong focus on the intake of individual nutrients and their relationship to health outcomes. More recently, methods to assess overall diet quality have been employed as an attempt to capture not only the quantity of nutrient intake but also dietary diversity and how well an individual’s dietary pattern adheres to dietary guidelines [22]. However, while validation of these scores is often undertaken against micronutrient intake, the association between diet quality scores and chronic disease biomarkers is less consistent [9,22]. In the present study, associations were noted between dietary quality scores and ‘metabolic’ health markers (abdominal obesity and HDL-cholesterol), but not other ‘cardiovascular’ health markers (blood pressure and heart rate).

Consistent with a recent finding from the CHARGE consortium [23], we noted an inverse association between quality of dairy intake and total cholesterol, but interestingly, this was not observed when total dairy intake was examined as a percentage of total energy. In line with Australian dietary guidelines, the ARFS dairy score calculation allocates a higher score for low-fat milk [9], as dairy fats are a source of saturated fat and there has been concern about adverse effects of this saturated fat intake on cholesterol levels and subsequent cardiovascular risk. However, there remains a lack of clear evidence that the consumption of low-fat dairy products is associated with lower cardiovascular risk when compared to high-fat dairy [24,25].

In middle-aged women, greater dietary quality of vegetable intake (ARFS vegetable score, which encompasses both variety and quantity of vegetable intake) was associated with fewer Medicare (health service) claims [26]. However, dietary quality using this measure was not found to be related to the subsequent development of obesity in a previous study [27]. In the present cohort, total diet quality, as well as the quality of dietary intake of fruits and grains, but not vegetables, was associated with a marker of abdominal obesity (waist to hip ratio).

Sugar-sweetened beverage consumption among those aged 65 years and over in the most recent Australian National Health Survey was 16% for women and 22.4% for men [28]. Thus, consumption of sugar-sweetened beverages in this cohort was higher than national average intakes, consistent with greater consumption by those living outside major metropolitan areas and in areas of greater socioeconomic disadvantage [19]. The cluster of cardiometabolic risk factors associated with intake of sugar-sweetened beverages in the present study were those that form the criteria for metabolic syndrome. This is consistent with findings from cross-sectional studies that have suggested an association between sugar-sweetened beverage intake and metabolic syndrome, although this has not consistently been observed in prospective studies [29].

### Strengths and Limitations

The diet quality scores used in this study (ARFS) are relatively newly developed, and this is one of the first studies to have examined ARFS and cardiometabolic disease risk markers. However, this was a cross-sectional analysis; thus, causality cannot be inferred. Nutrition or dietary epidemiology has some well-known limitations in terms of sources of error: (1) with diet being time-varying (e.g., due to seasonal, health, or economic factors), and (2) omission of foods (e.g., because dietary instruments rely on memory, epidemiological scale instruments may not capture all foods, or bias conferred by tendencies to misreport foods perceived as either ‘unhealthy’ or ‘healthy’) [30]. Of the dietary assessment tools available to researchers and clinicians, FFQs are less expensive and have a low participant burden, thus validated FFQs are often the most practical option for large-scale studies. While FFQs tend to give higher values relative to food diaries or 24 h recalls, FFQs are better able to capture seasonably consumed foods and capture usual or habitual intake. Comparison to Australian national data is limited by the differences in dietary assessment methodology, with an FFQ used in this study compared to a 24-h dietary recall in the Australian National Health Survey [19]. Furthermore, the participants were not a truly random sample of the source population, as the sample was over-represented by people with a history of cardiovascular disease. There were other potential sources of bias relating to dietary intake and cardiometabolic risk that were not accounted for in these models, including non-cardiovascular medication use, cultural factors, living alone, income, work status, and other comorbidities.

## 5. Conclusions

Among older, regionally-dwelling adults, potentially modifiable dietary risk factors for cardiometabolic disease are common, namely inadequate intakes of fibre and folate, and excessive sodium intake. Women have higher dietary quality scores for total diet, vegetable, fruit, and dairy intake compared to men. Public health strategies aiming to reduce intake of sugar-sweetened beverages may be of particular benefit in this population.

## Figures and Tables

**Table 1:** Demographic and cardiometabolic risk factor characteristics.

	Men (*n* = 256)	Women (*n* = 202)	*p*
Age (years)	70 ± 9	73 ± 7	<0.001
Area level disadvantage: n (%) first IRSAD quintile	164 (64.1)	141 (69.8)	0.482
School education to year 10 or below	73 (28.6)	99 (49.3)	<0.001
Meeting physical activity guidelines (self-reported)	123 (48.4)	91 (45.3)	0.510
Body Mass Index (kg/m^2^)	29.9 ± 5.0	30.3 ± 6.5	0.36
Waist Circumference (cm)	108.9 ± 13.4	100.3 ± 15.1	<0.001
Waist to Hip ratio	1.04 ± 0.06	0.90 ± 0.07	<0.001
Diabetes	53 (20.8)	29 (14.6)	0.110
History of CVD	131 (49.6)	95 (45.6)	0.408
Current smoker	19 (7.5)	14 (7.0)	0.096
Systolic/Diastolic Blood Pressure (mmHg)	133/72 ±17/11	135/72 ± 19/11	0.878
Heart rate (bpm)	63 ± 11	67 ± 11	0.003
Total cholesterol (mmol/L)	4.36 ± 0.99	4.85 ± 0.98	<0.001
HDL cholesterol (mmol/L)	1.21 ± 0.33	1.47 ± 0.37	<0.001
LDL cholesterol (mmol/L)	2.33 ± 0.88	2.58 ± 0.89	0.004
HbA1c (%)	6.1 ± 1.1	5.9 ± 0.8	0.128
Estimated glomerular filtration rate (eGFR) (mL/min/1.73 m^2^)	71.2 ± 15.6 ^a^	71.3 ± 15.7 ^b^	0.955

Values given as Mean ± SD or n (% of reporting population) unless otherwise specified. *p:* differences between men and women. Smaller sample size for estimated glomerular filtration rate ^a^ *n* = 173, ^b^ *n* = 141.

**Table 2:** Dietary characteristics for men and women.

	Men *(n = 256)*	Women *(n = 202)*	*p*
Energy (kJ/day)	9727 (9326–10128)	7989 (7537–8441)	<0.001
ARFS-total (score/73)	29.0 (27.8–30.3)	33.1 (31.6–34.5)	<0.001
ARFS-Vegetable (score/21)	11.4 (10.8–12.0)	13.5 (12.8–14.2)	<0.001
ARFS-Fruit (score/12)	4.7 (4.4–5.1)	5.5 (5.1–5.9)	0.004
ARFS-Grain (score/13)	4.0 (3.8–4.2)	4.2 (4.0–4.5)	0.198
ARFS-Meat (score/7)	2.7 (2.5–2.9)	2.9 (2.7–3.0)	0.230
ARFS-Alternate Protein (score/6)	1.7 (1.5–1.8)	1.9 (1.7–2.0)	0.071
ARFS-Dairy (score/11)	3.6 (3.4–3.8)	4.0 (3.7–4.2)	0.011
ARFS-Spreads-Sauces (score/2)	0.8 (0.7–0.9)	0.8 (0.7–0.9)	0.736
ARFS-Water (score/1)	0.3 (0.3–0.4)	0.5 (0.4–0.6)	0.001
Carbohydrate (% of total energy)	45.4 (44.5–46.3)	42.7 (41.7–43.7)	<0.001
Protein (% of total energy)	18.7 (18.3–19.1)	20.5 (20.0–21.0)	<0.001
Fat (% of total energy)	32.5 (31.8–33.2)	34.3 (33.5–35.0)	0.001
Saturated fat (% of total energy)	14.1 (13.7–14.5)	15.0 (14.5–15.4)	0.008
Polyunsaturated fat (% of total energy)	3.8 (3.7–3.9)	4.0 (3.8–4.1)	0.032
Confectionery (% total energy)	5.6 (4.9–6.2)	4.6 (3.8–5.3)	0.047
Baked sweet products (% total energy)	6.1 (5.5–6.7)	5.9 (5.2–6.5)	0.294
Takeaway foods (% total energy)	7.2 (6.7–7.8)	5.9 (5.3–6.5)	0.002
Alcoholic beverage (% of total energy)	5.0 (4.0–5.9)	4.0 (3.0–4.0)	0.177
Sugar-sweetened drinks (% total energy)	3.6 (3.0–4.2)	1.7 (1.1–2.4)	<0.001
Soft drinks (% consuming ≥ 1/week)	48 (18.8)	22 (10.9)	0.252
Sugar-sweetened drinks (%consuming≥ 1/week)	76 (29.9)	38 (18.8)	0.008
Fresh fish (% consuming ≥ 1/week)	55 (21.5)	71 (35.2)	0.01
Canned fish (% consuming ≥ 1/week)	78 (30.5)	75 (37.2)	0.379
Crumbed/battered fish (% consuming ≥ 1/week)	50 (19.6)	50 (24.7)	0.036
Sodium (mg/day)	2329 (2218–2440)	1984 (1857–2109)	<0.001
Potassium (mg/day)	3927 (3757–4097)	3526 (3334–3718)	0.003

Values given as adjusted means (95% CI) or n (%) in those with complete dietary data. Means are adjusted for age and education. The denominator of ARFs scores vary as indicated (score/denominator) [9]. *p:* Significance of sex-differences.

**Table 3:** Prevalence of inadequate nutrient intakes ^#^.

	Men (*n* = 256)	Women (*n* = 202)
Protein	21 (8.2)	8 (4.0)
Fibre	148 (57.8)	101 (50.2)
Folate	104 (40.6)	100 (49.8)
Vitamin A	31 (12.1)	17 (8.5)
Vitamin C	5 (2)	1 (0.5)
Thiamine	29 (11.3)	25 (12.4)
Riboflavin	16 (6.3)	12 (6)
Sodium	155 (60.5)	85 (42.3)
Potassium	128 (50)	57 (28.4)
Magnesium	88 (34.4)	40 (19.9)
Calcium	103 (40.2)	106 (52.7)
Iron	10 (3.9)	5 (2.5)
Zinc	92 (35.9)	12 (6)

Values given as n (%). ^#^ Proportions NOT meeting age- and gender-specific estimated average requirements (EARs) [16], except for sodium (% above suggested dietary target) and potassium (% below adequate intake).

**Table 4:** Australian Recommended Food Scores and modifiable cardiometabolic risk factors.

	Age- and Gender-adjusted (Model 1) ^a^	Multivariable-Adjusted ^b^ (Model 2)	Model 2 excl. Potential Misreporting ^c^
**Waist:hip**			
ARFS total	−0.001 (−0.001, 0.000) **	−0.001 (−0.001, 0.000) *	−0.001 (−0.001, 0.000) *
ARFS vegetable	−0.001 (−0.003, 0.000) *	n.s.	n.s.
ARFS fruit	−0.003 (−0.005, −0.001) **	−0.002 (−0.004, 0.000) *	−0.003(−0.005, −0.001) *
ARFS grain	−0.006 (−0.009, −0.003) ***	−0.004 (−0.007, −0.001) *	−0.004(−0.007,−0.001) **
ARFS alt. protein	−0.005 (−0.009, 0.000)*	n.s.	n.s.
**HDL cholesterol**			
ARFS vegetable	0.008 (0.002, 0.015) **	n.s.	0.007 (0.000, 0.013) *
ARFS meat	0.045 (0.023, 0.068) ***	0.037 (0.015, 0.059) **	0.044 (0.021, 0.067) ***
**eGFR**			
ARFS meat	1.210 (0.002, 2.418)	1.365 (0.164, 2.566) *	1.305 (0.022, 2.587) *
ARFS alt. protein ^d^	1.339 (0.001, 2.677)	1.342 (0.021, 2.664) *	n.s.
**Total cholesterol**			
ARFS dairy	−0.080 (−0.129, −0.030) **	−0.067 (−0.109, −0.024) **	−0.073 (−0.118,−0.028) ***

Values are Unstandardised Beta Coefficients (95% CI), n.s. = not significant. alt. protein= non-meat protein sources. ^a^ Model 1 adjusted for age and gender (*n* = 458). ^b^ In Model 2, all variables are adjusted for age, gender, smoking, physical activity, education, and BMI, except waist:hip, which is adjusted for age, gender, smoking, physical activity, education and diabetes, and total cholesterol, which is additionally adjusted for lipid-lowering therapy use. ^c^ Model 2, excluding those with total dietary energy <2000 kJ and >15000 kJ (total *n* = 432). ^d^ Significant interaction between dietary scores and gender, *p* < 0.01. * *p* < 0.05, ** *p* < 0.01, *** *p* ≤ 0.001.

**Table 5:** Fish intake and modifiable cardiometabolic risk factors.

	Age- and Gender-Adjusted (Model 1) ^a^	Multivariable Adjusted ^b^ (Model 2)	Model 2 excl. Potential Misreporting ^c^
**Waist:hip**			
Crumbed/battered fish	0.003 (0.000, 0.006)	0.003 (0.000, 0.006) *	0.004 (0.001, 0.007) **
**HDL cholesterol**			
Canned fish	0.022 (0.009, 0.035) ***	0.023 (0.010, 0.036) ***	0.026 (0.013, 0.040) ***
**eGFR**			
Fresh fish	0.966 (0.239, 1.692) **	1.001 (0.273, 1.730) **	1.128 (0.373, 1.184) **
**HbA1c**			
Canned fish	−0.038 (−0.076,−0.001) *	−0.032 (−0.064,−0.001) *	−0.030 ( −0.63, 0.002)

Values are Unstandardised Beta Coefficients (95%CI). ^a^ Model 1 adjusted for age and gender (*n* = 458). ^b^ In Model 2 all variables are adjusted for age, gender, smoking, physical activity, education, and BMI, except waist:hip, which is adjusted for age, gender, smoking, physical activity, education, and diabetes. ^c^ Model 2 excluding those with total dietary energy <2000 kJ and >15000 kJ. * *p* < 0.05, ** *p* < 0.01, *** *p* ≤ 0.001.

**Table 6:** Beverage intakes and modifiable cardiometabolic risk factors.

	Age- and Sex-Adjusted (Model 1) ^a^	Multivariable Adjusted ^b^ (Model 2)	Multivariable Adjusted ^c^ (Model 3)	Model 3 Excluding Potential Misreporting ^d^
**Waist:hip**				
Sugar-sweetened beverages (%E)	0.002 (0.001, 0.003) **	0.002 (0.001, 0.003) **	0.002 (0.001, 0.003) **	0.002 (0.001, 0.003) **
Soft drink (number consumed/day)	0.010 (0.002, 0.017) *	n.s.	n.s.	n.s.
**HbA1c**				
Sugar-sweetened beverage (%E)	0.032 (0.014–0.051) ***	0.035 (0.019–0.050) ***	0.035 (0.019–0.050) ***	0.029 (0.013–0.045) ***
Soft drink (number consumed/day)^e^	0.262 (0.143, 0.380) ***	0.174 (0.076, 0.272) ***	0.178 (0.078, 0.277) ***	0.159 (0.054, 0.264) **
Diet soft drink (times consumed)	0.058 (0.033, 0.083) ***	0.029 (0.008, 0.050) **	0.029 (0.009, 0.050) **	0.030 (0.009, 0.051) **
**HDL cholesterol**				
Sugar-sweetened beverage (%E)	−0.011 (−0.017,−0.004) ***	−0.009 (−0.015,−0.002) ***	−0.008 (−0.015,−0.001) *	−0.008 (−0.015, −0.001) *
Soft drink (number consumed/day)	−0.093 (−0.135,−0.051) ***	−0.062 (−0.104,−0.021) **	−0.061(−0.103,−0.019) **	−0.071 (−0.115, −0.027) **
Diet soft drink intake	−0.011 (−0.020, −0.002) *	n.s.	n.s.	n.s.
**eGFR**				
Sugar-sweetened beverage (%E)	−0.419 (−0.748, −0.091) *	−0.400 (−0.744, −0.055) *	−0.363 (−0.709, −0.017) *	−0.396 (−0.759, −0.032) *
Soft drink (number consumed/day)	−3.802 (−6.099,−1.504) ***	−3.110 (−5.464, −0.756) *	−2.794 (−5.184, −0.404) *	−3.351 (−5.909, −0.792) *
Diet soft drink intake	−0.484 (−0.941, −0.028) *	n.s.	n.s.	n.s.

Values are Unstandardised Beta Coefficients (95% CI), n.s. = not significant. Beverage intake as % total energy intake (%E), or number consumed. ^a^ Model 1 adjusted for age and sex (*n* = 458). ^b^ In Model 2, all variables are adjusted for age, sex, smoking, physical activity, education, and BMI, except waist:hip, which is adjusted for age, sex, smoking, physical activity, education, and diabetes. ^c^ In Model 3, all variables are adjusted for age, sex, smoking, physical activity, education, diabetes, BMI, and ARFS (total), except waist:hip, which is adjusted for age, sex, smoking, physical activity, education, diabetes, and ARF total score. ^d^ Model 3 excluding those with total dietary energy <2000 kJ and >15000 kJ (*n* = 432). ^e^ Significant interaction between dietary scores and gender, *p* = 0.012
